# N_2_-foam-assisted CO_2_ huff-n-puff process for enhanced oil recovery in a heterogeneous edge-water reservoir: experiments and pilot tests

**DOI:** 10.1039/d0ra09448j

**Published:** 2021-01-04

**Authors:** Hongda Hao, Jirui Hou, Fenglan Zhao, Handong Huang, Huaizhu Liu

**Affiliations:** China University of Petroleum Beijing 102249 China haohongda90@126.com +86 17601007653; Drilling & Production Technology Research Institute, Jidong Oilfield Company, CNPC Tangshan Hebei 063000 China

## Abstract

The CO_2_ huff-n-puff process is an effective method to enhance oil recovery; however, its utilization is limited in heterogenous edge-water reservoirs due to the severe water channeling. Accordingly, herein, a stable N_2_ foam is proposed to assist CO_2_ huff-n-puff process for enhanced oil recovery. Sodium dodecyl sulfate (SDS) and polyacrylamide (HPAM) were used as the surfactant and stabilizer, respectively, and 0.3 wt% of SDS + 0.3 wt% of HPAM were screened in the laboratory to generate a foam with good foamability and long foam stability. Subsequently, dynamic foam tests using 1D sand packs were conducted at 65 °C and 15 MPa, and a gas/liquid ratio (GLR) of 1 : 1 was optimized to form a strong barrier in high permeable porous media to treat water and gas channeling. 3D heterogeneous models were established in the laboratory, and N_2_-foam-assisted CO_2_ huff-n-puff experiments were conducted after edge-water driving. The results showed that an oil recovery of 13.69% was obtained with four cycles of N_2_-foam-assisted CO_2_ injection, which is twice that obtained by the CO_2_ huff-n-puff process. The stable N_2_ foam could temporarily delay the water and gas channeling, and subsequently, CO_2_ fully extracted the remaining oil in the low permeable zones around the production well. Pilot tests were conducted in 8 horizontal wells, and a total oil production of 1784 tons with a net price value (NPV) of $240 416.26 was obtained using the N_2_-foam-assisted CO_2_ huff-n-puff process, which is a profitable method for enhanced oil recovery in heterogenous reservoirs with edge water.

## Abstract 2

Edge water and gas can be temporarily delayed with the assistance of stable N_2_ foam. The oil recovery enhanced by N_2_-foam-assisted CO_2_ huff-n-puff was twice that by pure CO_2_ huff-n-puff.

## Graphical abstract

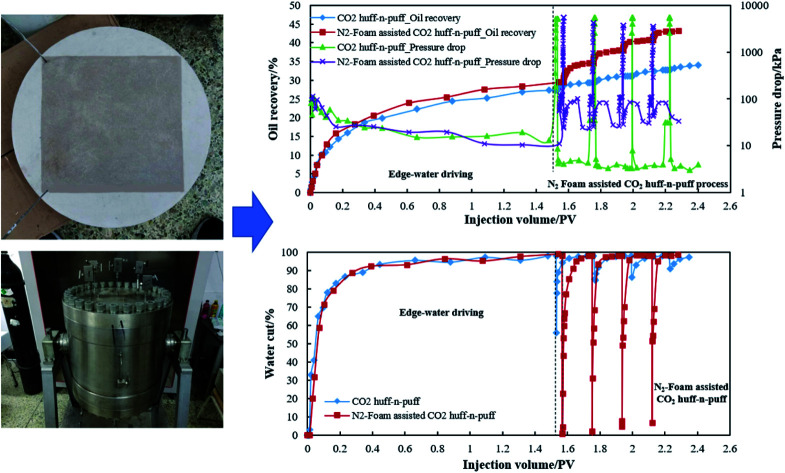

## 1. Introduction

Edge-water-driving reservoirs are widely distributed in China, which account for important proportions of geological reserves of crude oil.^1–4^ The existence of edge water has both advantages and disadvantages for the development of oilfields. On one hand, edge water can provide energy for oil production and maintain the formation pressure. On the other hand, the invasion of edge water can cause a quick increase in the water cut and a low oil recovery efficiency.^5,6^ North Gaoqian Block, located in the north of Bohai Bay, China, is a heavy oil reservoir with a sufficient edge-water aquifer. The permeability of this reservoir is in the range from 118 × 10^−3^ μm^2^ to 12 392 × 10^−3^ μm^2^, which indicates that serious heterogeneity exists in this reservoir. The reservoir began to be developed using horizontal wells in 2004, and more than 12 × 10^4^ tons of crude oil was obtained annually using edge-water driving. However, due to the serious edge-water channeling, the water cut increased sharply to more than 90% within 5 years, and the oil recovery was less than 15% up to 2010. Therefore, appropriate techniques need to be conducted to enhance the oil recovery (EOR) in this heterogeneous reservoir.

CO_2_-EOR is a promising technique to enhance heavy oil recovery, which has been successfully used in many countries.^7–12^ As one of the CO_2_-EOR methods, the CO_2_ huff-n-puff process is usually conducted in a single well, and three stages are included in this process as follows: (1) injection stage: CO_2_ is injected into the formation through an operation well. (2) Soaking stage: the well is shut-in for a period to allow CO_2_ to be dissolved with the formation oil. (3) Production stage: the operation well is then reopened for production. The CO_2_ huff-n-puff process can be immiscible, near-miscible and miscible, which depends on the minimum miscibility pressure (MMP) between CO_2_ and oil. When the pressure is less than the MMP, the oil recovered by CO_2_ is mainly through oil swelling, viscosity reduction, light components of oil extraction, and relative permeability of water and gas reduction.^13–17^ When the pressure approaches or exceeds the MMP, the interfacial tension (IFT) between CO_2_ and oil can be sharply reduced to form a near-miscible or miscible CO_2_ huff-n-puff process, which will drastically enhance the oil recovery.^18,19^

The CO_2_ huff-n-puff process has been applied in North Gaoqian Block, China since 2010, and more than 16 × 10^4^ tons of crude oil was recovered by CO_2_ until the end of 2017. However, problems were also found during the operation process. For example, the oil recovery gradually decreased after multicycles of gas injection, and the water cuts of single wells were usually above 99% after cycling due to edge-water channeling. Heterogeneity not only provides channels for edge water, it also severely affects the displacement efficiency of CO_2_. Due to the serious heterogeneity, CO_2_ mostly flows along high permeable layers or channels, leaving plenty of crude oil in the low permeable layers. Thus, treatments need to be done to deal with water and gas channeling during the CO_2_ huff-n-puff process.

The used of foam was proposed as an EOR method to reduce water and gas permeability in the 1960s.^20,21^ As a colloidal system, foam is usually made of a discontinuous gas dispersed in a continuous liquid phase, where gas bubbles are separated by thin liquid films called lamellae.^22–24^ Since gas is wrapped in bubbles in the liquid phase, the apparent viscosity is several orders of magnitude greater than either gas or liquid, which then effectively inhibits viscous fingering and enhances the oil recovery.^25,26^ When foam is injected into heterogenous reservoirs, it will first enter the high permeable zones with higher porosity and better connectivity. As the foam transports through the formations, high flow resistance will be caused in high permeable zones due to the Jamin effect. Consequently, the successive displacing agent will be impelled to displace the oil in the low permeable zones.^27–29^ Recently, laboratory experiments have shown that foam can even be used to plug severe water or gas channels such as fractures, and the oil recovery enhanced by foam injection can reach 25–30%.^30–32^

Although significant EOR factors can be obtained using foams, most of the foam injections are operated during the flooding process, and only a few studies have been conducted in the foam-assisted huff-n-puff process. When assisting the CO_2_ huff-n-puff process, the foam should have special properties as follows. (1) The surfactant must be pH tolerant to ensure good foamability due to the CO_2_ environment. As a type of anionic surfactant, sodium dodecyl sulfate (SDS) is broadly used in sandstone reservoirs, which has advantages of foaming, thermal stability and low cost.^33–35^ (2) A stabilizer needs to be added to generate a more stable foam for water and gas plugging. Adding a polyacrylamide (HPAM) additive can effectively increase the apparent viscosity and enhance the stability of foam.^36^ (3) Excellent pressure maintenance should be achieved with the minimal usage of foam in the huff-n-puff process. N_2_ is a type of non-condensate gas, which can effectively build-up the formation pressure. Furthermore, many researchers have found that N_2_ foam is usually stronger and more stable compared to CO_2_ foam.^37–39^ Thus, herein, stable N_2_ foam was used to assist the CO_2_ huff-n-puff process for EOR.

To generate a stable N_2_ foam to assist the CO_2_ huff-n-puff process, SDS and HPAM were selected as the surfactant and stabilizer, respectively, and their concentrations were firstly optimized in the laboratory *via* the evaluation of foamability and foam stability. Then, dynamic foam tests using 1D sand-packs were conducted to evaluate the plugging mechanisms for water and gas channeling. 3D heterogeneous models were established in the laboratory, and N_2_-foam assisted CO_2_ huff-n-puff experiments were conducted to study the EOR effects with the assistance of N_2_-foam plugging. Finally, pilot tests were also introduced, and a simplified economic analysis was performed to evaluate the economic benefits resulting from the N_2_-foam-assisted CO_2_ huff-n-puff process.

## 2. Experiments

**Fig. 1:**
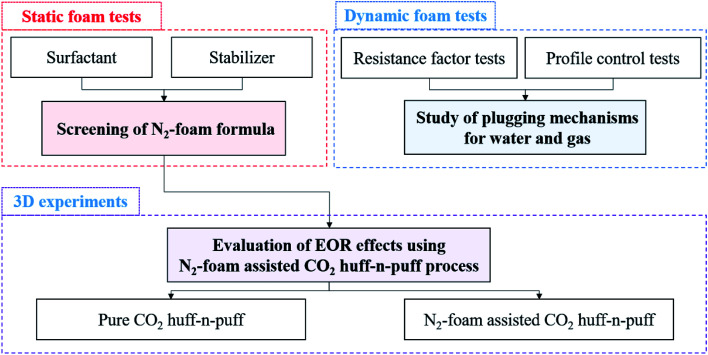
Flow chart for N_2_-foam-assisted CO_2_ huff-n-puff experiments.

Three main experiments were designed to study the mechanisms and EOR effects using the N_2_-foam-assisted CO_2_ huff-n-puff process. Static foam tests were performed to screen the concentration of surfactant and stabilizer, dynamic foam tests were performed to study the plugging mechanisms for water and gas channeling, and 3D experiments were performed to evaluate the EOR effects of the N_2_-foam-assisted CO_2_ huff-n-puff process. A flow chart for the experiments conducted herein is shown in Fig. 1.

### 2.1. Materials

Sodium dodecyl sulfate (SDS, active concentration of 99%) was used as the surfactant and polyacrylamide (HPAM, molecular weight of 20 million Daltons) was used as the stabilizer. Both the surfactant and polymer were obtained from Beijing Hengju Chemical Group, Co. LTD., China. Nitrogen (N_2_, purity >99.99 mol%) was used for the generation of foam, and carbon dioxide (CO_2_, purity >99.999 mol%) was used for the huff-n-puff process, which were both provided by Beijing Jinggao Gases Co. LTD., China. The formation oil and water were collected from the reservoir block. The density of the formation oil was 0.89 g cm^−3^, its viscosity was 58.21 mPa s and the gas/oil ratio was 42.42 m^3^/m^3^ under the formation conditions (65 °C, 15 MPa). The compositions of the formation oil are presented in Table 1. The salinity of the formation water was 1937 mg L^−1^.

**Table 1:** Composition of the formation oil

Component	Mol%	Component	Mol%
CO_2_	0.000	nC_4_	0.256
N_2_	1.800	iC_5_	0.026
C_1_	20.310	nC_5_	0.033
C_2_	6.648	C_6_	0.066
C_3_	1.152	C_7+_	69.325
iC_4_	0.384	Total	100

### 2.2 N_2_-foam evaluation experiments

#### 2.2.1 Static foam tests

The foamability and foam stability were tested using the Waring blender method at ambient temperature and atmospheric pressure.^40–43^ The solution was firstly prepared with a mixture of formation water, SDS and HPAM. The concentration of SDS ranged from 0.1 wt% to 0.6 wt%, and the concentration of HPAM ranged from 0.1 wt% to 0.5 wt%. 200 mL of solution was stirred for 60 s at a speed of 10 000 rpm to generate foam, and then the foam was poured into a graduated cylinder to measure the foam volume (*V*_F_) and half-life time (*t*_1/2_). The foam volume (*V*_F_) was used to reflect the foamability of the chemical agent, while the half-life time (*t*_1/2_) was used to reflect the foam stability (measured as the time used for half-volume dewatering). Then, a foam composite index (FCI) was used to evaluate the foam performance comprehensively,^44,45^ which was calculated as FCI = 0.75*V*_F_ × *t*_1/2_. A suitable formula of foaming agent was screened according to the foam volume (*V*_F_), half-life time (*t*_1/2_), and FCI index, and then utilized in the following experiments and pilot tests.

#### 2.2.2 Dynamic foam tests

Single and dual sand packs were used to evaluate the dynamic performance of the N_2_ foam. The length of the single sand pack was 30 cm, its diameter was 2.5 cm, and its permeability ranged from 500 × 10^−3^ μm^2^ to 8000 × 10^−3^ μm^2^. A high permeable (500 × 10^−3^ μm^2^) sand pack and a low permeable (3000 × 10^−3^ μm^2^) sand pack were parallelly connected to form a dual sand pack model. The other physical parameters of the models are listed in Tables 2 and 3.

**Table 2:** Physical parameters of the single sand packs

No.	Experiment	Apparent volume/mL	Pore volume/mL	Porosity/%	Permeability/×10^−3^ μm^2^
1	RF *vs.* GLR	147.54	55.5	37.59	3431
		147.31	52.2	35.43	3118
		146.83	50.5	34.37	2775
		147.07	54.6	37.12	3276
		147.42	55.4	37.61	3303
2	RF *vs.* permeability	146.97	41.8	28.41	469
		147.16	45.1	30.65	1231
		146.83	50.5	34.37	2775
		147.22	52.8	35.88	5314
		147.39	55.1	37.39	8253

**Table 3:** Physical parameters of dual sand pack model

No.	Sand pack	Apparent volume/mL	Pore volume/mL	Porosity/%	Permeability/×10^−3^ μm^2^
1	Low permeability	147.13	40.4	27.44	535
2	High permeability	147.25	52.5	35.66	3431

**Fig. 2:**
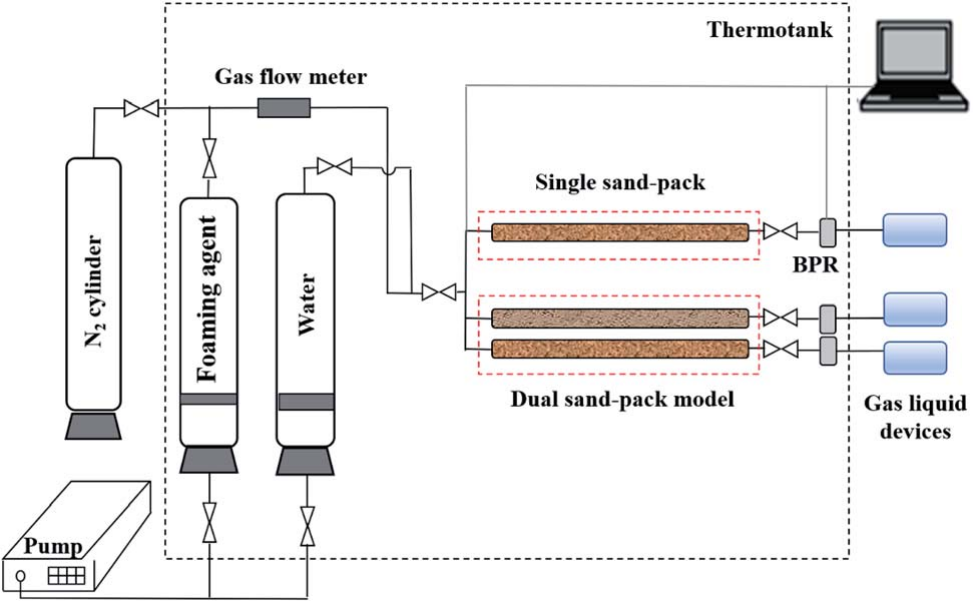
Schematic diagram showing the dynamic N_2_-foam experiments.

A schematic diagram of the dynamic foam tests is shown in Fig. 2. The equipment mainly consisted of four parts, including an injection system, a displacement system, a production system and a data acquisition system. For the injection system, the formation water and foaming agents were stored in containers, which were driven by an injection pump. N_2_ was provided by a nitrogen cylinder, and was controlled using a gas flow meter. For the displacement system, the single sand pack or dual sand pack model was placed in a thermotank to simulate the formation conditions. Back pressure regulation (BPR) was used in the production system to maintain the pressure as formation pressure, and the produced water and gas were collected by a gas–liquid device. The injection pressure, production pressure and differential pressure drop were recorded by the data acquisition system.

The resistance factors (RFs) of different gas/liquid ratios (GLRs) were firstly measured using the single sand packs. The RF can be used as an index to evaluate the plugging ability of N_2_ foam,^46–48^ which is defined as RF = (Δ*p*_foam_/Δ*p*_water_)_at same rate_, where Δ*p*_foam_ is the differential pressure drop for foam injection, kPa, and Δ*p*_water_ is the differential pressure drop for water injection, kPa. The experimental procedures are detailed as follows. (1) Preparation: a single sand pack with a permeability of 3000 × 10^−3^ μm^2^ was used in this section. After the sand pack was saturated with formation water, the porosity of the sand pack was calculated as the ratio of pore volume (PV, equal to saturated water volume) to apparent volume. (2) Water injection: the temperature of the thermotank was set as 65 °C, and the pressure of the BPR was set as 15 MPa. Formation water was injected into the sand pack at a constant rate of 0.5 mL min^−1^, and then terminated when the differential pressure drop of water (Δ*p*_water_) was steady. (3) Foam injection: N_2_ and the foaming agent were alternately injected into the sand pack with a flow rate of 0.5 mL min^−1^. The GLR value varied as 1 : 2, 1.5 : 1, 1 : 1, 1 : 1.5 and 1 : 2, and was terminated when the pressure drop of the foam (Δ*p*_foam_) was steady. The RF value was then calculated according to the pressure drop, and a suitable GLR was determined for the subsequent experiments.

The RF value of the N_2_ foam in different permeable sand packs was then measured with the optimal GLR value. Sand packs with a permeability of 500 × 10^−3^ μm^2^, 1000 × 10^−3^ μm^2^, 3000 × 10^−3^ μm^2^, 5000 × 10^−3^ μm^2^ and 8000 × 10^−3^ μm^2^ were used in this section. The experimental procedures for preparation, water injection and foam injection were the same as mentioned above. Also, the plugging ability of N_2_ foam for different permeable porous media was evaluated by comparing the RF values obtained in the different permeable sand packs.

A profile control experiment using N_2_ foam was also conducted in a dual sand pack model. After the experimental preparation, 0.50 PV of water was injected into the model, followed by 0.05 PV of N_2_ foam, then successive water was injected into the model until the N_2_ foam was totally displaced from the model. The injection rate of water and foam was set as 0.5 mL min^−1^, and the liquid production rates of high (*Q*_h_) and low (*Q*_l_) permeable sand packs were recorded separately throughout the experimental process. The profile control ability of N_2_ foam can be evaluated by comparing the changes in *Q*_h_ and *Q*_l_.

### 2.3 3D experiments for N_2_-foam-assisted CO_2_ huff-n-puff

**Fig. 3:**
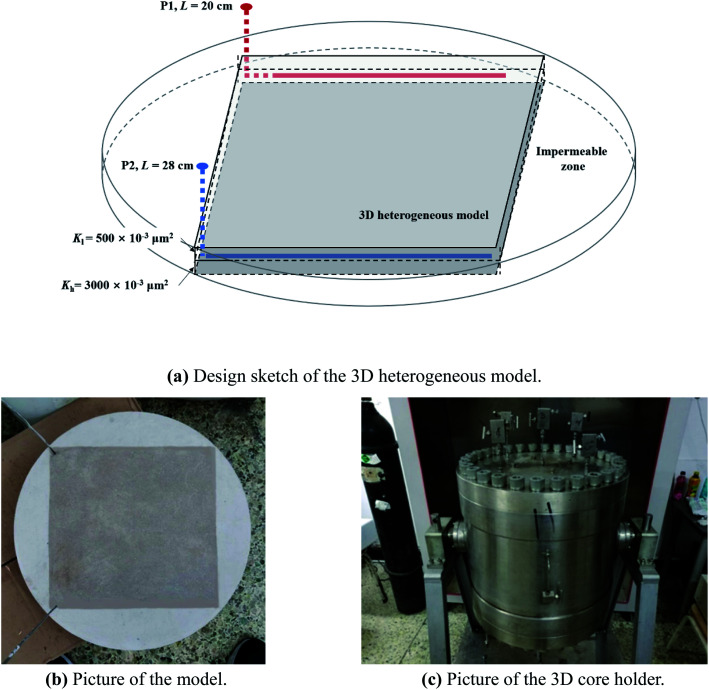
3D physical model for the N_2_-foam-assisted CO_2_ huff-n-puff process.

The N_2_-foam-assisted CO_2_ huff-n-puff experiment was conducted in a 3D physical model in the laboratory. The model was heterogeneous with two layers, where the permeability of the upper layer (*K*_l_) was 500 × 10^−3^ μm^2^, the permeability of the sublayer (*K*_h_) was 3000 × 10^−3^ μm^2^, and the permeability contrast was 6. The model size was 30 × 30 × 4.5 cm^3^, as shown in Fig. 3, and the other parameters of the 3D model are listed in Table 4. Well P2 was designed for edge-water injection with a length of 28 cm, and well P1 was designed as a producer with a length of 20 cm. Four impermeable zones were fabricated beside the four sides of the model in order to fit the 3D core holder, and no fluids could exchange between the permeable zone and impermeable zones (as shown in Fig. 3(a) and (b)). The 3D core holder (as shown in Fig. 3(c)) was specially utilized for the high temperature and high pressure experiments with an operation temperature in the range of 0–100 °C and operation pressure in the range of 0–30 MPa.

**Table 4:** Physical parameters of the 3D experimental models

No.	Experimental scheme	Apparent volume/mL	Pore volume/mL	Porosity/%	Permeability/×10^−3^ μm^2^	Initial oil saturation/%
1	CO_2_ huff-n-puff	4059	1110	27.34	500/3000	65.47
2	N_2_-foam assist CO_2_ huff-n-puff	4041	1030	25.49		63.26

**Fig. 4:**
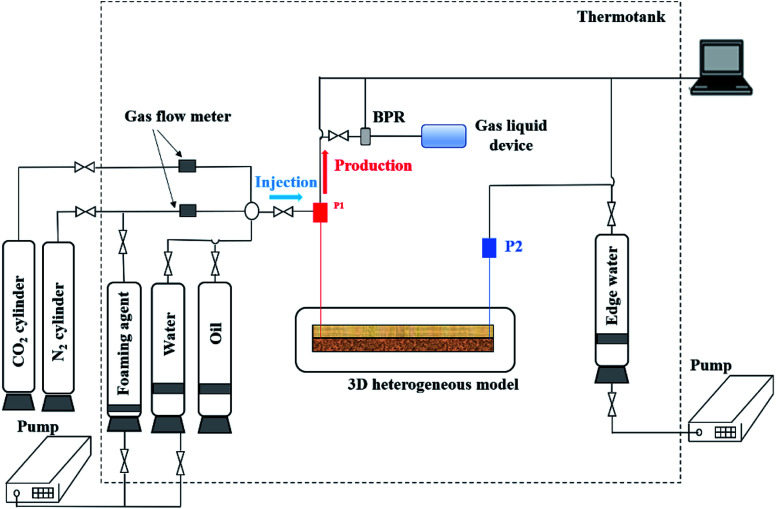
Schematic diagram of the N_2_-foam-assisted CO_2_ huff-n-puff process.

Fig. 4 shows a flow chart of the N_2_-foam-assisted CO_2_ huff-n-puff experiment. Similar to the sand pack equipment, the injection system, displacement system, production system and data acquisition system were also included in the 3D experimental apparatus. For the injection system, the formation water, formation oil and foaming agents were stored in containers, and N_2_ and CO_2_ were provided by nitrogen and CO_2_ cylinders. For the displacement system, the 3D model was placed in a thermotank to simulate the reservoir conditions. For the production system, a BPR was used to maintain the formation pressure, and the produced water, oil and gas were collected by a gas–liquid device. The injection pressure, production pressure and differential pressure drop were also recorded by the data acquisition system. Moreover, an edge-water injection system was specially designed to simulate the edge-water injection, which was driven by another injection pump.

To evaluate the EOR effects of the N_2_-foam-assisted CO_2_ huff-n-puff process, a comparative experiment of the CO_2_ huff-n-puff process was also conducted without the assistance of N_2_-foam. Scenario 1 was utilized for the CO_2_ huff-n-puff experiment, and the experimental procedure is detailed as follows. (1) Preparation: the 3D model was held by the core holder and saturated with formation water and oil. The porosity, initial water and oil saturation were then calculated according to the injection volume of water and oil. Then, the model was placed in the thermotank with a temperature of 65 °C, and the BPR pressure of P1 was set as 15 MPa. (2) Edge-water driving: edge water was injected through P2 at an injection rate of 0.5 mL min^−1^, and P1 was opened for production simultaneously. After the water cut of P1 reached 98%, P1 and P2 were shut, and the edge-water driving process was terminated. (3) CO_2_ huff-n-puff: P2 remained shut, and P1 was opened for CO_2_ injection. When the injection volume of CO_2_ reached 900 mL (under standard conditions), P1 was shut, and the gas injection stage was terminated. After 12 h of soaking time, P2 was reopened to continue edge-water injection, and P1 was reopened for production. When the water cut of P1 reached 98% again, P1 and P2 were shut, and one cycle of CO_2_ huff-n-puff was finished. Three more cycles were conducted on the model, and then the experiment was finished. The production of oil, water and gas, and the pressure were recorded during the experimental process.

The N_2_-foam-assisted CO_2_ huff-n-puff experiment was conducted after edge-water driving in Scenario 2. The experimental procedures for the preparation and edge-water driving are the same as Scenario 1, while the procedure for the N_2_-foam-assisted CO_2_ huff-n-puff process is detailed as follows. (1) N_2_-foam injection: a slug of N_2_ foam was pre-injected into the model before CO_2_ injection. N_2_ and the foaming agents were alternately injected through P1 at an injection rate of 0.5 mL min^−1^. Considering the poor compressive properties of the foaming agents, P2 was changed into an production well, and remained open during the injection stage. After 10 mL of N_2_ and 10 mL of foaming agent were injected, P1 and P2 were shut. (2) CO_2_ huff-n-puff: P2 remained shut, and P1 was opened for CO_2_ injection. When the injection volume of CO_2_ reached 800 mL, P1 was shut, and the CO_2_ injection stage was terminated. After 12 h of soaking time, P2 was reopened to continue edge-water injection, and P1 was reopened for production. When the water cut of P1 reached 98% again, P1 and P2 were shut, and one cycle of N_2_-foam-assisted CO_2_ huff-n-puff process was finished. Three more cycles were conducted on the 3D model, and then the experiment was completed. The production of oil, water and gas, and the pressure were recorded during the experimental process.

## 3. Results and discussion

### 3.1 Static and dynamic performance of N_2_ foam

**Fig. 5:**
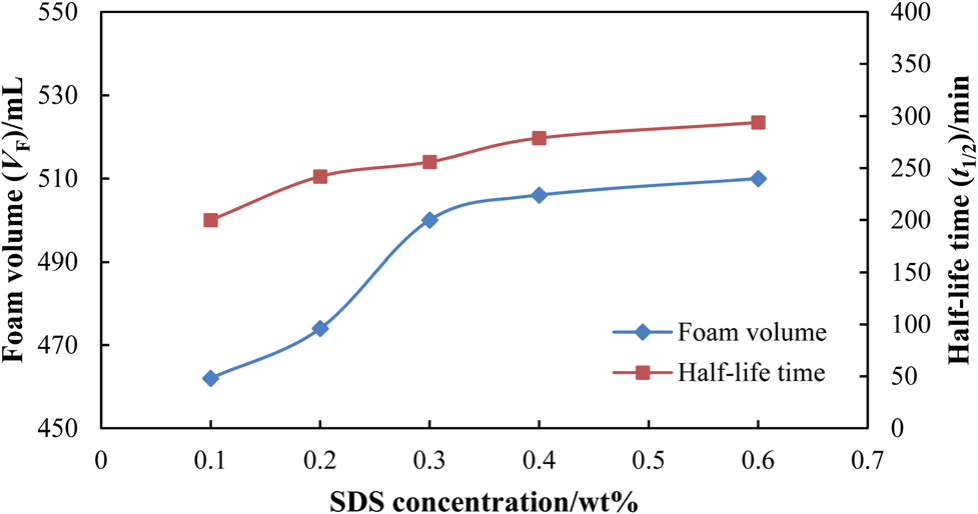
Static foam performance with different SDS concentrations.

The surfactant is an essential ingredient to generate foam, and thus its concentration was firstly screened at ambient temperature and atmospheric pressure. Then, 0.3 wt% of stabilizer (HPAM) was added to the foaming agent, and the static foam performance of SDS with different concentrations was evaluated, as shown in Fig. 5. The foam volume increased rapidly when the SDS concentration increased from 0.1 wt% to 0.3 wt%, which indicates that a high concentration of surfactant is beneficial for foamability. When the foam volume increased slightly when the SDS concentration was higher than 0.3 wt%, the foam volume remained at a high value, and excessive surfactant had little influence on the foamability. The half-life time also increased with an increase in the concentration of SDS, and a more stable foam was generated with a higher concentration of surfactant.

**Fig. 6:**
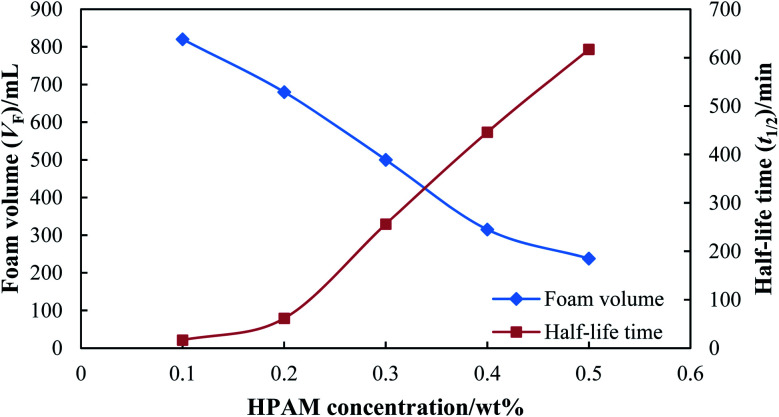
Static foam performance with different HPAM concentrations.

Fig. 6 shows the static foam performance with different HPAM concentrations, where the concentration of SDS was kept constant at 0.3 wt%. Compared with the influence of SDS concentration, HPAM concentration had a greater influence on the foam performance. The half-life time increased significantly from 16.5 min to 617 min when the HPAM concentration increased from 0.1 wt% to 0.5 wt%, which indicates that a more stable foam can be formed with a higher concentration of stabilizer. However, the foam volume decreased sharply from 820 mL to 238 mL when the HPAM concentration increased from 0.1 wt% to 0.5 wt%. A higher HPAM concentration has a negative effect on foamability, and thus a suitable concentration of stabilizer should be screened considering both foamability and foam stability.

**Fig. 7:**
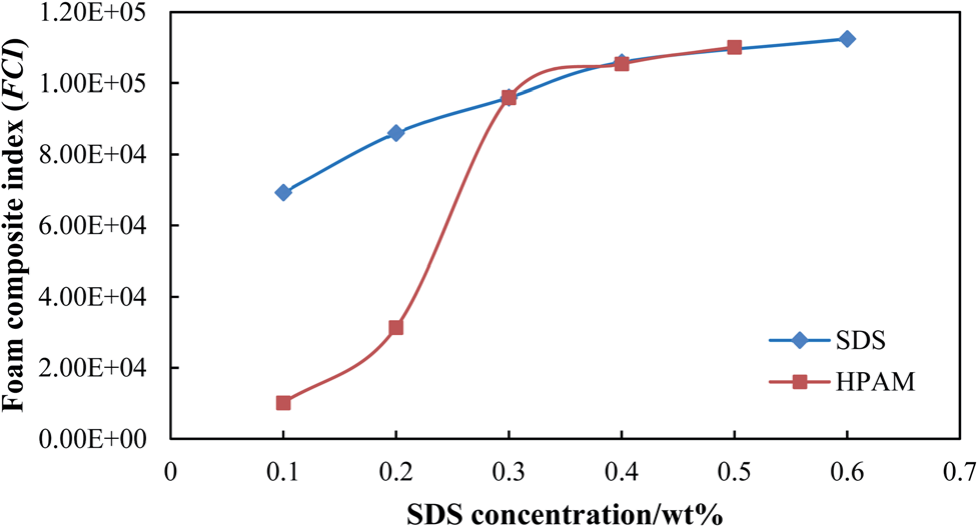
Foam composite index (FCI) for different foaming agents.

Fig. 7 shows the foam composite index (FCI) for different foaming agents. The FCI of SDS increased with an increase in the concentration of SDS, and remained at a much higher value compared with that of HPAM (HPAM concentration <0.3 wt%). Since the FCI of SDS is mainly dominated by foam volume, a concentration of SDS equal to or higher than 0.3 wt% is suitable for foamability. Thus, 0.3 wt% of SDS was used in the following experiments and pilot tests considering cost saving. The FCI of HPAM increased rapidly when the HPAM concentration increased from 0.1 wt% to 0.3 wt%, where the foam showed excellent foamability but a poor stability. Although the FCI of HPAM remained at a higher value when the HPAM concentration was higher than 0.3 wt%, the foam showed an excellent stability but a poor foamability. Thus, the concentration of stabilizer was set as 0.3 wt% with comprehensive consideration of foamability and stability.

**Fig. 8:**
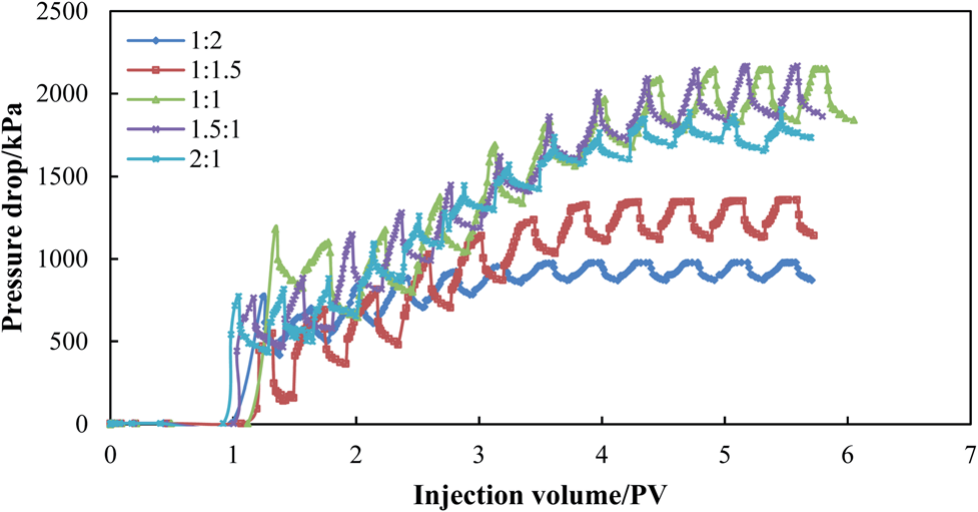
Pressure drop of N_2_ foams with different gas/liquid ratios.

The dynamic foam performance was then evaluated using the foaming agent consisting of 0.3 wt% SDS and 0.3 wt% HPAM. The foaming agent and N_2_ were alternately injected into single sand packs under the formation conditions (65 °C and 15 MPa), and the pressure drop for the different gas/liquid ratios (GLRs) is shown in Fig. 8. Specifically, 0.10 PV of water was pre-injected before N_2_ foam injection, and the pressure drop of water was about 6 kPa. The pressure drop increased gradually with an increase in the N_2_-foam injection volume, and fluctuated by alternating the gas/liquid. Since N_2_ and the foaming agent were alternately injected into the sand packs, a barrier was firstly built by the viscous foaming agent. When the following slug of N_2_ was injected, a small portion of gas phase was trapped by the barrier, which then delayed the expansion of the gas phase. With the alternating of N_2_ slug and liquid slug, an increasing amount of gas was trapped in the porous media, which then caused an increase in the pressure drop. However, the trapping effect was also influenced by the GLR ratio. The equilibrium pressure drop was used to compare the pressure buildup by N_2_ foams with different GLRs, which is an average value of the gas and liquid pressure drop. The N_2_ foam with a GLR value of 1 : 2 or 1 : 1.5 achieved the lowest pressure drop of less than 1400 kPa, where N_2_ saturation as too low to form a stable barrier, and the pressure buildup was mainly due to the flow of polymer and surfactant in the porous media. When the GLR value was equal to or more than 1 : 1, N_2_ and the foaming agent could be mixed sufficiently to form a strong barrier for water and gas channeling, which then caused a pressure of up to more than 2100 kPa. When the GLR value reached 2 : 1, although a similar pressure gradient could also be built in the initial injection period, gas channeling occurred through the sand pack due to excessive N_2_ injection, leading to a slight decrease in the equilibrium pressure drop. Table 5 lists the resistance factor (RF) of the N_2_ foams with different GLR values. When the GLR value was equal to or more than 1 : 1, the RF value was calculated to be greater than 300. Since the N_2_ foam with a GLR of 1 : 1 achieved the highest RF value of 341.98, the GLR value was set as 1 : 1 for the following experiments.

**Table 5:** Resistance factors (RFs) of N_2_ foams measured in single sand packs

No.	Permeability/×10^−3^ μm^2^	GLR	RF
1	3000 (±500)	1 : 2	159.82
2		1 : 1.5	212.77
3		1 : 1	341.98
4		1.5 : 1	340.31
5		2 : 1	301.46
6	469	1 : 1	59.73
7	1231		114.10
8	2775		341.98
9	5314		494.38
10	8253		571.67

**Fig. 9:**
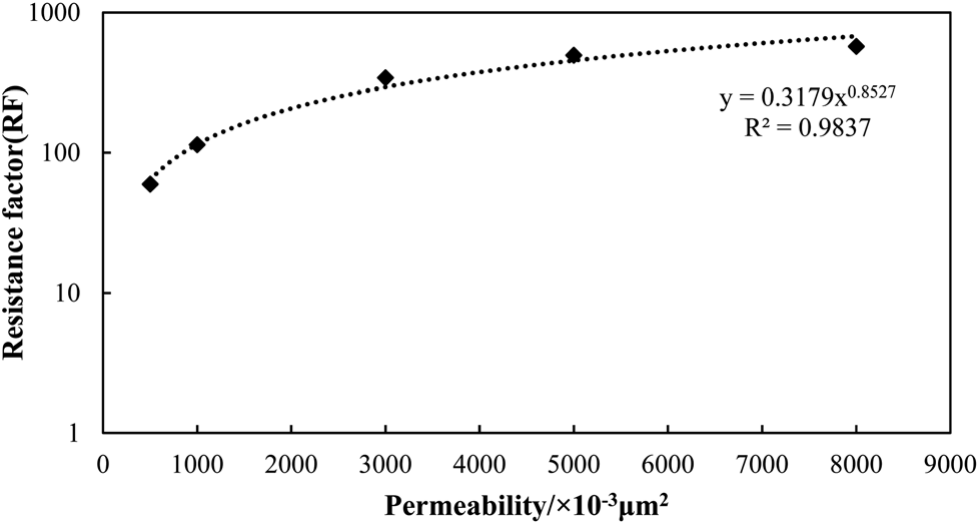
Resistance factor of N_2_ foam in different permeable sand packs.

The RF values of the N_2_ foams in the different permeable sand packs were also measured, and the results are shown in Fig. 9. It can be seen that the RF value exhibits an exponential increase with the change in permeability. When the permeability increased from 500 × 10^−3^ μm^2^ to 8000 × 10^−3^ μm^2^, the RF value increased dramatically from 59.73 to 571.67. This observation is consistent with the result reported by Jian *et al.*^49^ Since the shearing force decreases with an increase in the pore throat size, the gas saturation trapped in the foaming agent can be fully maintained in higher permeable porous media. N_2_ foam tends to form a stronger barrier in the higher permeable zone, which is beneficial for water and gas plugging in heterogeneous reservoirs.

**Fig. 10:**
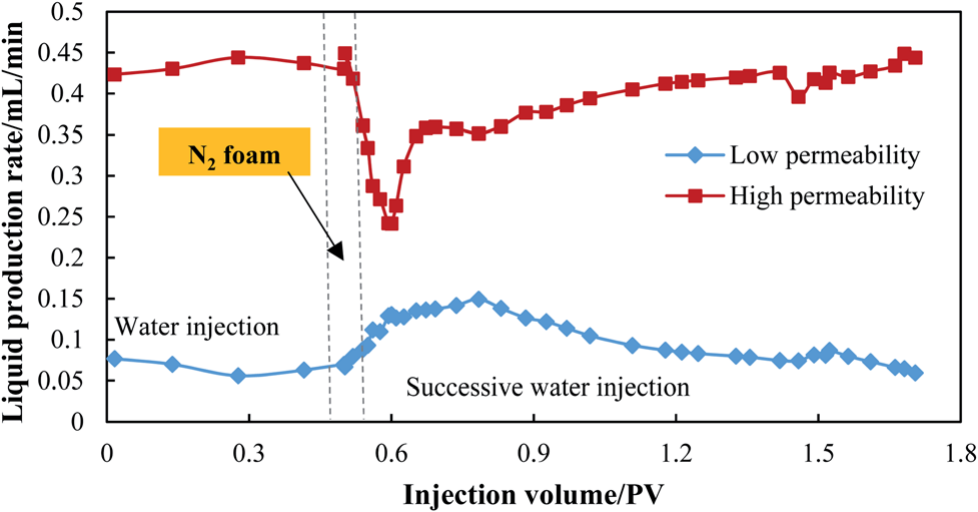
Liquid production performance of N_2_ foam treatment in dual sand pack.

A profile control experiment using N_2_ foam was also conducted using a dual sand pack, and the result is shown in Fig. 10. During the water injection process, the liquid production rate of the high permeable sand pack was 0.43 mL min^−1^, which accounts for 86.75% of the total production rate. The water mainly flowed along the high permeable sand pack, leading to a low sweep efficiency in the low permeable sand pack. After 0.05 PV of N_2_-foam plugging, the liquid production rate of the high permeable sand pack could be reduced to 0.24 mL min^−1^, which is almost half the rate before foam plugging. After the high permeable sand pack was effectively plugged by foam, the water and gas were then diverted to flow in the low permeable sand pack, and the production rate of the low permeable sand pack doubled from 0.07 mL min^−1^ to 0.13 mL min^−1^. Furthermore, this profile improvement lasted for 1.15 PV of successive water injection until the N_2_ foam was totally displaced from the sand packs, which indicates that the barrier formed by N_2_ foam has a long validity for water and gas plugging. The high strength, ability of fluid diversion and long validity for profile control with minimal usage (0.05 PV) make the use of the foam possible to treat water/gas channeling and assist the CO_2_ huff-n-puff process.

### 3.2 EOR effects of N_2_-foam-assisted CO_2_ huff-n-puff

To study the EOR effect of N_2_-foam-assisted CO_2_ huff-n-puff in a heterogenous edge-water reservoir, 3D experiments were conducted in the laboratory, and the results are presented in Table 6. A pure CO_2_ huff-n-puff process was conducted after edge-water driving in Scenario 1 for comparison. During the edge-water driving process, water firstly breakthrough with an injection volume of just 0.01 PV. Then, the water cut increased sharply to 90% after 0.4 PV of edge-water injection. When the water cut of P1 reached 98%, the oil recovery of edge-water driving was 27.32% and 29.40% for these two models, respectively. Due to the heterogeneity, the injected edge water mostly flowed through the high permeable layer, and the oil recovery was mostly attributed to the oil displaced from the high permeable layer. After the channeling of the edge water, plenty of oil remained in the low permeable layer. For the near-wellbore area of well P1, the oil in the low permeable zone could even be unswept by the edge water.

**Table 6:** 3D experimental results of N_2_-foam-assisted CO_2_ huff-n-puff

No.	Period	Cycle no.	N_2_ foam (GLR = 1 : 1)/mL	CO_2_ volume (surface)/mL	Oil volume/mL	Oil recovery factor/%
1	Edge-water driving	—	—	—	198.5	27.32
	CO_2_ huff-n-puff	1	—	900	14.8	2.03
		2	—	900	12.8	1.76
		3	—	900	11.4	1.57
		4	—	900	10.2	1.40
2	Edge-water driving	—	—	—	191.6	29.40
	N_2_-foam assisted CO_2_ huff-n-puff	1	20	800	33.6	5.15
		2	20	800	21.6	3.31
		3	20	800	18.2	2.79
		4	20	800	15.9	2.44

**Fig. 11:**
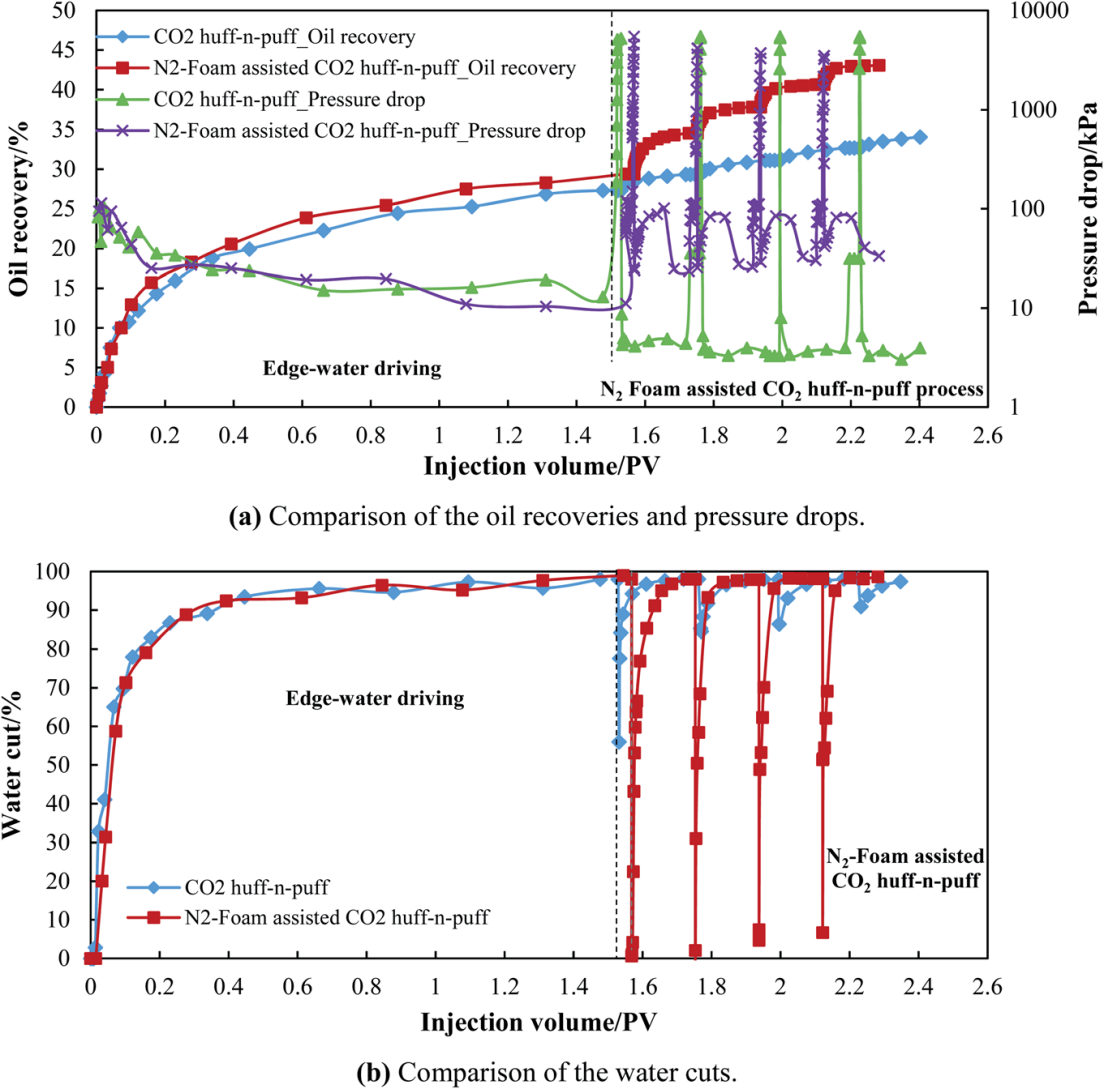
Performance of CO_2_ huff-n-puff and N_2_-foam-assisted CO_2_ huff-n-puff processes.

For Scenario 1, four cycles of CO_2_ huff-n-puff processes were conducted with an injection volume of 900 mL CO_2_ (under standard conditions) for each cycle. The pressure drop increased from 12.97 kPa to more than 5 MPa after the CO_2_-injection stage; however, it decreased sharply to less than 5 kPa when P1 was reopened for production (as seen in Fig. 11(a)). The water cut dropped sharply from 98% to 60–90% in the initial stage of production, and then increased rapidly to more than 95% (as seen in Fig. 11(b)). The changes in water cut correspond to the changes in pressure drop. CO_2_ is expected to extract the oil remaining in the low permeable layer, and it indeed contacted with the oil after a sufficient soaking period. However, since oil and CO_2_ were produced from P1 quickly in the initial production stage, the edge water flowed back again through the high permeable layer. After water and gas channeling occurred, a portion of CO_2_ was still trapped in the model, which indicates that the CO_2_-EOR effect is severely affected by the serious channeling of water and gas. The oil recovery of CO_2_ injection was just 2.03%, 1.76%, 1.57%, and 1.40% for each cycle, and plugging treatments needed to be conducted to deal with the channeling along the high permeable layer.

For the N_2_-foam-assisted CO_2_ huff-n-puff process, 20 mL of foam was pre-injected into the model, followed by 800 mL of CO_2_, and the oil recovery was 5.15%, 3.31%, 2.79% and 2.44% for each cycle. The oil recovery enhanced by the N_2_-foam-assisted CO_2_ huff-n-puff was almost two times that by pure CO_2_ huff-n-puff. Fig. 11(a) and (b) show a comparison of the pressure drops and the water cuts for Scenario 1 and Scenario 2, respectively. Although a similar pressure drop was achieved after N_2_-foam-assisted CO_2_ injection, the pressure drop and the water cut showed a big difference during the production stage. It can be observed that the production stage can be further subdivided into three periods as follows.

(1) An oil and gas production period achieved by N_2_-foam plugging and CO_2_ extraction, which is short but very important to the oil increment. During this period, the pressure drop decreased rapidly from 3–5 MPa to less than 35 kPa, and the water cut dropped sharply to nearly zero. Since N_2_ foam was mostly injected into the high permeable layer, a strong barrier was temporarily built for the channeling of edge water. Then, the injected CO_2_ could sufficiently contact with the oil remaining in the low permeable zone near the P1 area, where the oil was almost unswept by the edge water. When P1 was reopened for production, this portion of oil was effectively extracted by the produced CO_2_, and almost no water was produced during this period.

(2) An oil, liquid and gas production period with N_2_ foam produced from P1 successively. During this period, the pressure drop increased again from less than 35 kPa to more than 80 kPa, and the water cut increased gradually to 95%. Since the barrier in the high permeable layer is broken due to the successive production of N_2_ and foaming agents, the edge water breakthroughs from P1 again. Oil, liquid and gas are produced simultaneously, and the oil recovery is slightly enhanced during this period.

(3) Edge-water channeling period after N_2_ foam is displaced from the model. During this period, the pressure drop decreased from more than 80 kPa to less than 35 kPa, and the water cut remained at a high level of 95–98%. Since the barrier was totally destroyed, the edge water channeled again through the high permeable layer, and a low amount of oil was produced during this period. Although a large portion of N_2_ foam was displaced from the model, a small portion of foaming agent could still remain in the high permeable layer. Consequently, the pressure drop during this period was higher than the pressure drop of the edge-water driving period.

The 3D experiments indicate that N_2_ foam can be used to assist the CO_2_ huff-n-puff process. The stable N_2_ foam can form a strong barrier to temporarily delay water and gas channeling, and subsequently CO_2_ can fully extract the crude oil remaining in the low permeable zone near the P1 area. The oil recovery enhanced by N_2_-foam-assisted CO_2_ huff-n-puff was almost twice that by pure CO_2_ huff-n-puff.

### 3.3 Pilot tests of N_2_-foam-assisted CO_2_ huff-n-puff

Several pilot tests of N_2_-foam-assisted CO_2_ huff-n-puff were conducted in North Gaoqian Block, Jidong Oil Field, China since 2015. Table 7 lists the results for 8 horizontal wells located in this block. After the edge-water driving and CO_2_ huff-n-puff process, about 400 tons of N_2_ foam and 200 000 m^3^ of CO_2_ were injected for each well. After an average soaking time of 30 days, the wells were reopened for production. An average oil production of 223 tons was obtained from a single well with an average valid period of 156 days. With the total injection volumes of 3288 tons N_2_ foam and 1 590 000 m^3^ CO_2_, the total oil production of 1784 tons was recovered from the heterogenous reservoir.

**Table 7:** Pilot results of N_2_-foam-assisted CO_2_ huff-n-puff

No.	Well no.	Foaming agent/ton	CO_2_ volume/m^3^ (SC)	Soaking time/day	Oil production increment/ton	Valid period/day	Net present value (NPV)/$
1	G104-5P76	320	200 000	30	176	127	15 442.74
2	G104-5P77	400	200 000	27	249	176	35 868.85
3	G104-5P78CP1	544	200 000	32	232	164	24 460.74
4	G104-5P85	404	200 000	26	240	138	33 272.99
5	G104-5P97	400	200 000	29	283	184	46 446.13
6	G104-5P101	300	160 000	40	210	123	33 522.20
7	G104-5P106CP1	540	250 000	25	225	163	14 816.73
8	G104-5P116	380	180 000	30	169	171	13 328.44
Average		411	198 750	30	223	156	27 049.73
Total		3288	1 590 000	—	1784	—	240 416.26

**Fig. 12:**
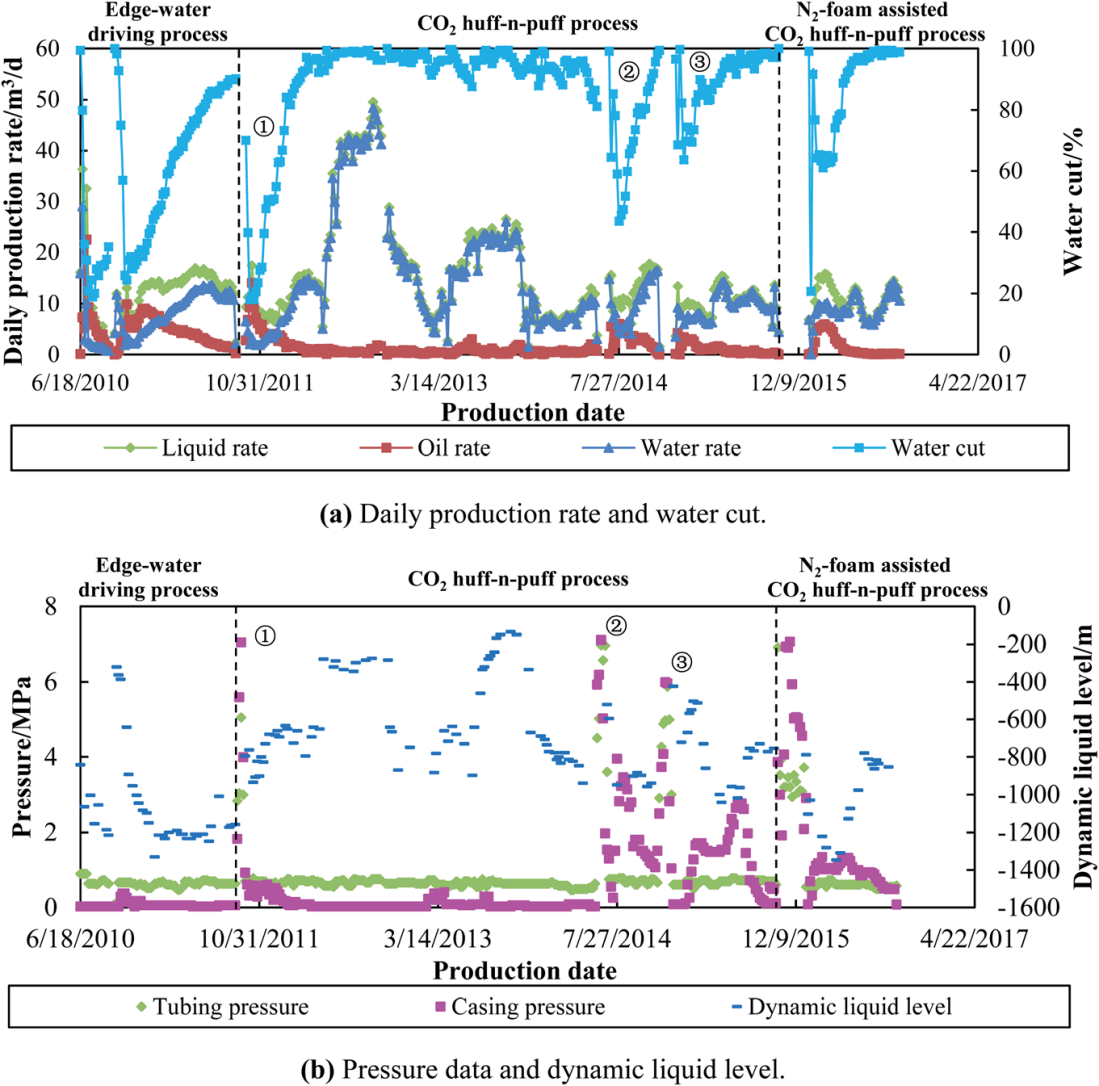
Production performance of G104-5P78C1 (N_2_-foam-assisted CO_2_ huff-n-puff).

Taking well G104-5P78C1 as a case study, this horizontal well is located near the edge-water aquifer, and was developed by natural energy on June 18, 2010. Although an oil production rate (daily) of 9.89 m^3^ per day was obtained in the initial period, it decreased rapidly to 1.21 m^3^ per day after one year of development. The water cut increased sharply to 90.11% due to the severe edge-water channeling, as shown in Fig. 12(a). Then, three cycles of CO_2_ huff-n-puff processes were conducted in this well for enhanced oil recovery. Specifically, 150 000 m^3^ of CO_2_ was injected into the formation for each cycle, and a total oil production of 862 tons was obtained after three cycles of CO_2_ injection. Since CO_2_ was injected when the water cut reached 90.11%, a portion of oil was unswept by the edge water, and still remained in both the high and low permeable zones. The CO_2_ huff-n-puff process could effectively reduce the water cut and enhance the oil recovery in the 1^st^ cycle. However, the EOR effects using CO_2_ injection were weakened in the 2^nd^ and 3^rd^ cycles, and operations need to be done to treat the heterogeneity of the reservoir.

Then, 544 m^3^ of N_2_ foam and 200 000 m^3^ of CO_2_ were injected into this well on Oct 14, 2015. After 32 days of soaking time, the well was reopened for production. The water cut dropped sharply from 100% to 20.47%, and the daily oil rate increased to 5.43 m^3^ per day in the initial production stage. The oil rate remained at 4–5 m^3^ per day, and the water cut remained between 60–65% for more than a month, which indicates that the high permeable zones were temporarily plugged by the N_2_ foam. After production for 164 days, 232 tons of crude oil was recovered using the N_2_-foam-assisted CO_2_ huff-n-puff process.

Fig. 12(b) shows the pressure data and dynamic liquid level for well G104-5P78C1. The tubing and casing pressure remained at less than 1 MPa during the edge-water driving period. During the 1^st^ cycle of CO_2_ injection, the tubing and casing pressure increased to 5 MPa and 7 MPa, respectively, and then dropped rapidly to less than 1 MPa again. For the 2^nd^ and 3^rd^ cycles of CO_2_ injection, the casing pressure increased to more than 6 MPa, and then slowly dropped to 1 MPa. This indicates that the formation energy was supplied by the injected gas, and the oil was displaced from the well by dissolved CO_2_ continuously. After N_2_ foam and CO_2_ were injected, the casing pressure increased to 7.06 MPa, and then dropped to 1 MPa when the well was reopened for production. The casing pressure remained at 1–1.5 MPa for more than 150 days, which also indicates that temporary plugging of water and gas channeling occurred by the N_2_-foam injection. The dynamic liquid level (DLL) is an index that can be used to reflect the channeling phenomenon. A higher DLL value means a more serious channeling. The DLL value was about −1200 m during the edge-water driving period, and then increased gradually to −800 m after three cycles of the CO_2_ huff-n-puff process. This high value of DLL was induced by the serious channeling along the high permeable zones. However, after the high permeable zones were plugged by N_2_-foam, the DLL value returned to the initial liquid level of −1200 m. The oil was enhanced, and the water cut was reduced correspondingly.

The production performance of well G104-5P78C1 is highly consistent with the performance observed in the 3D experiments. For the CO_2_ huff-n-puff experiments conducted in Scenario 1, it can be observed that with an increase in gas cycling, the oil recovery enhanced by CO_2_ injection decreased from 2.03% (1^st^ cycle) to 1.40% (4^th^ cycle), and the lowest water cut achieved by CO_2_ injection also increased gradually from 55.97% (1^st^ cycle) to 90.96% (4^th^ cycle). Similar observations were also found during the CO_2_ huff-n-puff processes performed in well G104-5P78C1. The oil production obtained by CO_2_ injections was 433 tons, 252 tons and 176 tons for the 1^st^, 2^nd^ and 3^rd^ cycles, and the lowest water cut was 18.02%, 43.60% and 63.66%, respectively. It can be predicted that if a 4^th^ cycle of pure CO_2_ huff-n-puff is conducted, the oil production would be less than 176 tons, and the lowest water cut would be higher than 63.66%. However, when a cycle of N_2_-foam assisted CO_2_ injection was performed after pure CO_2_ huff-n-puff, the oil increment was 232 tons, which is even higher than that obtained in the 3^rd^ cycle of pure CO_2_ injection. The water cut of the N_2_-foam-assisted CO_2_ injection also dropped to as low as 20.67%, and then remained between 60–65% for more than a month. This dramatic oil increment and water control are consistent with the experimental results obtained in Scenario 2, which means that N_2_-foam-assisted CO_2_ huff-n-puff is an effective method for enhanced oil recovery in heterogeneous reservoirs. With the assistance of N_2_-foam injection, the water and gas channeling can be temporarily delayed, and the oil remaining in the low permeable zones can be fully extracted by CO_2_ huff-n-puff process.

A simplified economic analysis of the N_2_-foam-assisted CO_2_ huff-n-puff process was performed by considering the income of the produced oil and the costs of the injected water, gas and foaming agent. Bouquet *et al.* proposed an economic evaluation of the EOR process using foam and CO_2_, where operation expenditures (OPEX) are mainly calculated, and capital expenditures (CAPEX) are not considered.^50^ The economics was quantified with the cash flow (CF), which is the difference between income and cost per month:

1 CF(*n*) = π_o_(*V*_o_^*n*^ − *V*_o_^*n*−1^) − [π_w_(*V*_w_^*n*^ − *V*_w_^*n*−1^) + π_g_(*V*_g_^*n*^ − *V*_g_^*n*−1^) + π_f_(*M*_f_^*n*^ − *M*_f_^*n*−1^)]

where *V*_o,w,g_^*n*^ is the cumulative volume of produced oil, injected water and gas at month *n* since the beginning of cycling, and *M*_f_^*n*^ is the cumulative mass of the foaming agents. π_o_ is the oil price, $ per bbl. Since the operations mentioned above were between the year of 2015 and 2016, π_o_ was set as 46.64$ per bbl. π_w_ is the injected water cost, $ per m^3^. π_g_ is the injected gas cost, $ per m^3^. π_f_ is the injected foaming agent cost, $ per kg. π_w_ is 0.66$ per m^3^, π_N_2__ is 0.1$ per m^3^, π_CO_2__ is 0.15$ per m^3^, and π_f_ is 4.82$ per kg for the operations in Jidong Oilfield, China.

Then, the net present value (NPV) was calculated as the discounted sum of the cash flow, which can be calculated as follows:2
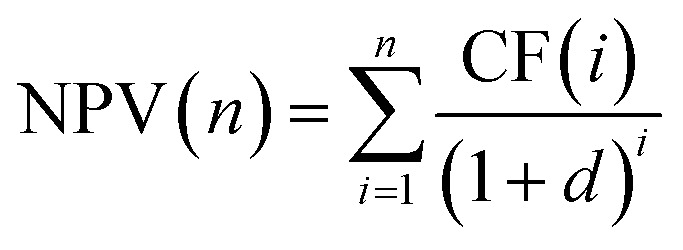
where the sum runs over month *i* = 1,…, *n*, and *d* represents the discount rate (set as 0.0067 here).

**Fig. 13:**
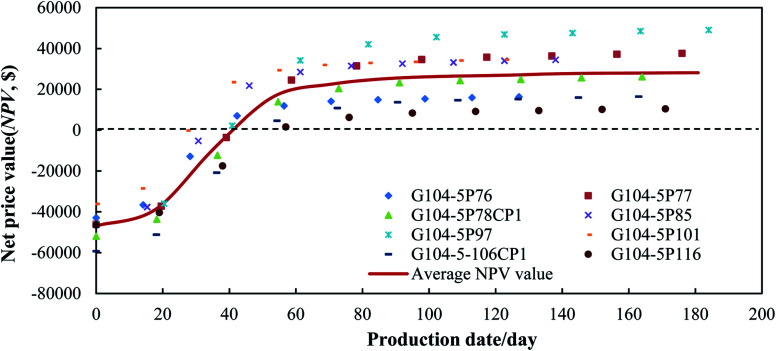
NPV values for pilot tests of N_2_-foam-assisted CO_2_ huff-n-puff process.

The calculated net present value (NPV) is listed in Table 7 and Fig. 13. After the operation of the N_2_-foam-assisted CO_2_ huff-n-puff process, the total NPV value was 240 416.26$ for 8 horizontal wells. Fig. 12 shows the changes in the NPV values with the production dates. Since all the N_2_, foaming agent and CO_2_ were injected before oil production, a one-time investment occurred with the costs of the water, gas and foaming agent. The initial NPV value ranged from −36 102.74$ to −59 284.94$ with an average value of −46 901.15$. The NPV value increased gradually with the oil production, and then reached a breakeven point (BEP) at around 45 days of oil production, where the incomes just covered the costs. The average oil increment at the BEP point was 150 m^3^ for a single well, then the produced oil brought net profits in the following production dates. At the end of the cycling process, the NPV value ranged from 10 475.52$ to 49 565.54$. The average NPV value for a single well was 28 145.51$ after cycling, which indicates that the N_2_-foam-assisted CO_2_ huff-n-puff process is a profitable technique for enhanced oil recovery in heterogeneous reservoirs with edge water.

## 4. Conclusions

A stable N_2_ foam with 0.3 wt% of sodium dodecyl sulfate (SDS) and 0.3 wt% of polyacrylamide (HPAM) as the surfactant and stabilizer, respectively, was screened in the laboratory. The dynamic foam tests showed that an alternation in the N_2_ and foaming agent with a gas/liquid ratio (GLR) value of 1 : 1 can form a strong barrier for water and gas channeling in porous media. A resistance factor (RF) of 341.98 was obtained in the high permeable layer, and a profile improvement with water and gas diversion was observed using N_2_-foam plugging. The 3D experimental results showed that the oil recovery enhanced by N_2_-foam-assisted CO_2_ huff-n-puff was twice that by CO_2_ huff-n-puff. With a temporarily delay of water and gas channeling achieved by the N_2_-foam, the oil remaining in the low permeable zones near the production well could be fully extracted by CO_2_ injection. Pilot tests were conducted in 8 horizontal wells, and a total oil production of 1784 tons with a total net price value (NPV) of 240 416.26$ was obtained using the N_2_-foam-assisted CO_2_ huff-n-puff process.

## Conflicts of interest

There are no conflicts to declare.

## Supplementary Material
